# Supplementary material for: Structure and Dynamics of the Integrin LFA-1 I-Domain in the Inactive State Underlie its Inside-Out/Outside-In Signaling and Allosteric Mechanisms
Source: Structure. 2015 Apr 7;23(4):745–53. doi: 10.1016/j.str.2014.12.020 (PMC4396694; doi:10.1016/j.str.2014.12.020)
Supplement: Document S1. Figures S1–S4 and Tables S1 and S2 [file mmc1.pdf]

Structure, Volume *23*

Supplemental Information

Structure and Dynamics of the Integrin LFA-1 I-Domain in the Inactive State Underlie its Inside-Out/ Outside-In Signaling and Allosteric Mechanisms

Predrag Kukic, Hoi Tik Alvin Leung, Francesco Bemporad, Francesco A. Aprile, Janet R. Kumita, Alfonso De Simone, Carlo Camilloni, and Michele Vendruscolo

Supplemental Figures

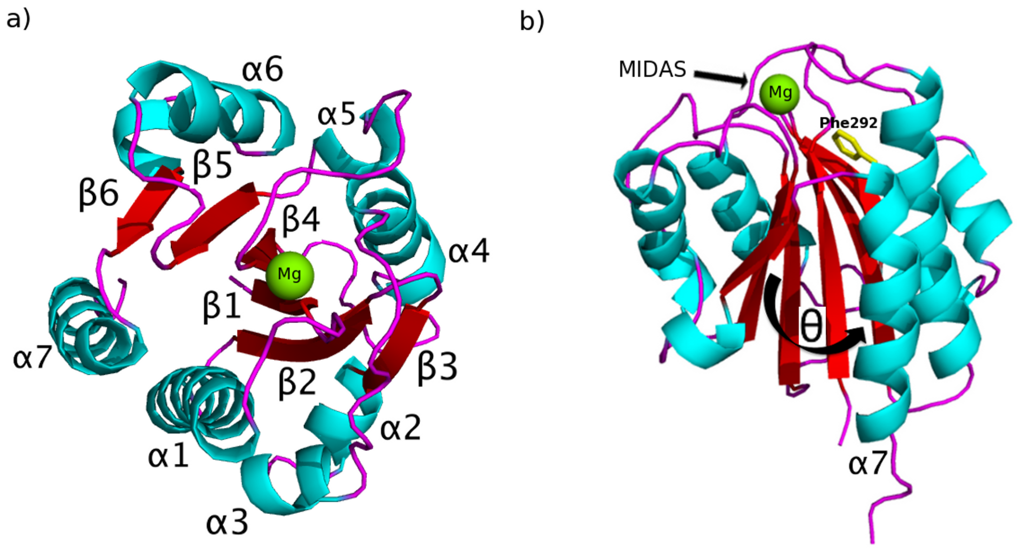

Figure S1, related to Figure 1. Secondary structure of the LFA-I domain. a) Cartoon view of the I-domain secondary structure elements taken from the low affinity (LA) state structure PBD ID: 1LFA. b) Definition of the angle θ between α-helix 7 and the hydrophobic core of the I-domain. The side chain of residue Phe292 in the LA state is locked inside the hydrophobic pocket that connects α-helix 7 and MIDAS, the so-called 'ratchet' pocket. In the intermediate affinity (IA) and high affinity (HA) states, the side chain of Phe292 is displaced from the ratchet pocket, which unlocks α-helix 7 and enables its movement along the side of the I-domain.

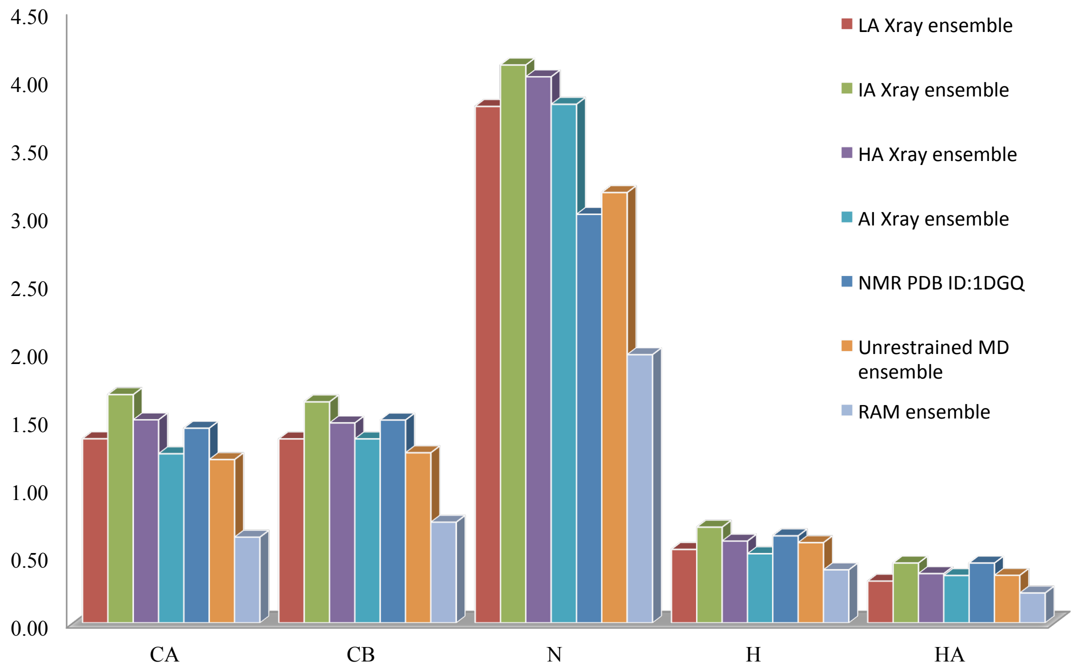

Figure S2, related to Figure 1. Bar chart representation related to Table S1.

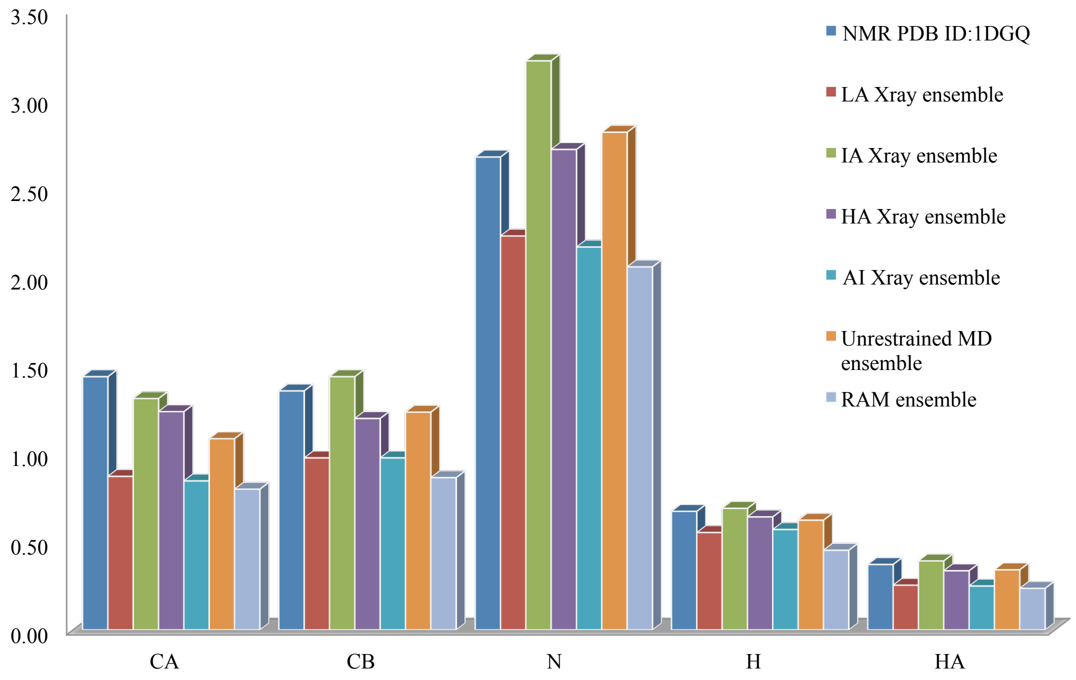

Figure S3, related to Figure 1. Bar chart representation related to Table S2.

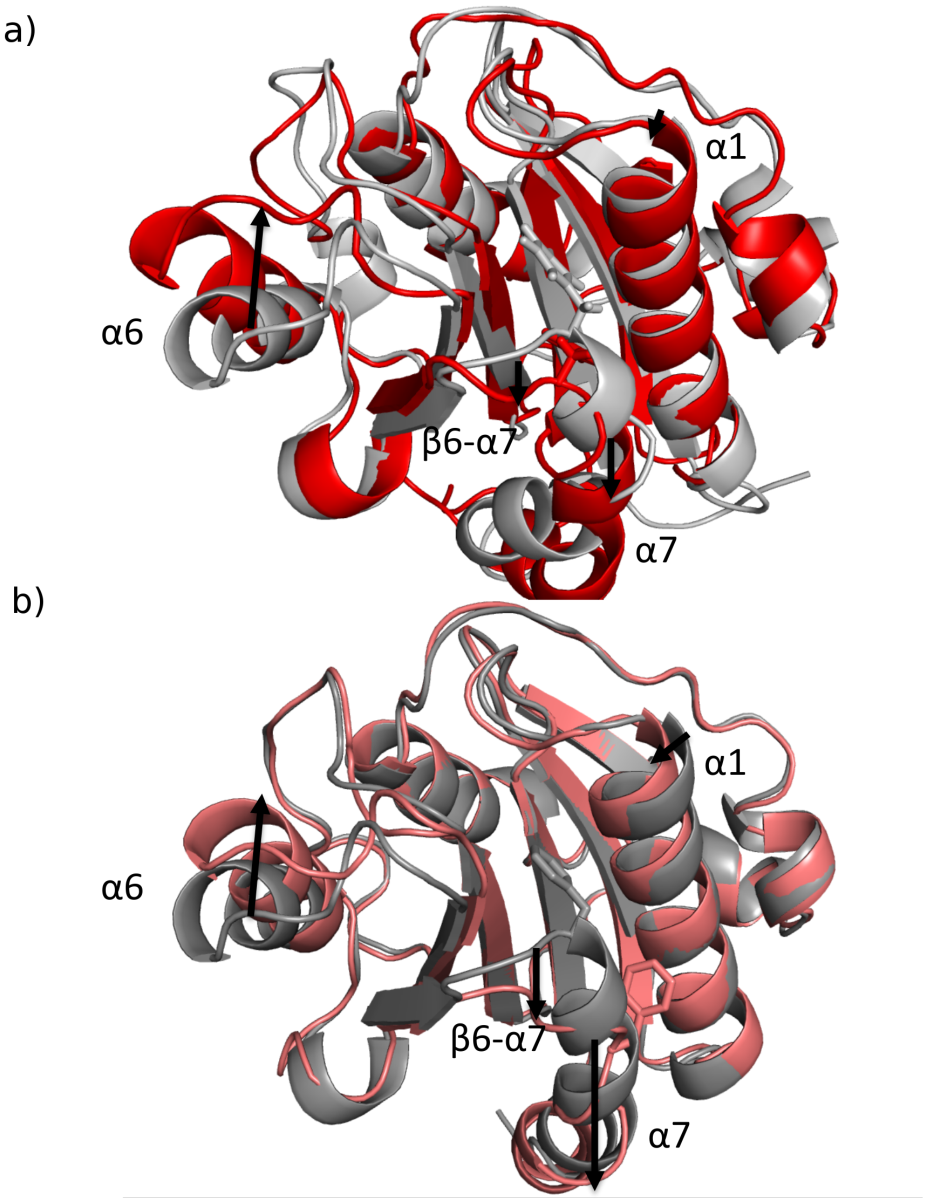

Figure S4, related to Figure 3. a) Superposition of the LA-like (gray) and IA-like states (red) from the RAM ensemble. Two representative structures with the lowest free energies in the RAM ensemble are superimposed using the hydrophobic core of the I-domain (residues 143-266). The arrows denote areas with the larges conformational differences: α-helix 7, loop between α-helix 7 and β-sheet 6, α-helices 1 and 6. b) Superposition of the representative crystallographic structures of the WT LFA in the LA (PDB ID: 3F74 depicted in gray) and HA states (PDB ID: 3TCX, depicted in light red). The areas of conformational differences are equivalent to those from the LA-like and IA-like structures.

Supplemental Tables

Table S1, related to Figure 1**.** Chemical shift-based comparison between the ensembles of crystallographic structures of LFA in the LA, IA, HA and AI states, the NOESY-derived structure PDB ID: 1DGQ, the unrestrained MD ensemble and the RAM ensemble. The comparison is made in terms of RMSD (ppm) between the experimentally measured chemical shifts and the values back-calculated from the ensembles using the CamShift method (Kohlhoff et al., 2009).

ensemble/chemical shifts	Cα	Cβ	N	H_N	Hα
NMR PDB ID:1DGQ	1.4	1.5	3.0	0.6	0.4
LA Xray ensemble	1.3	1.3	3.8	0.5	0.3
IA Xray ensemble	1.7	1.6	4.1	0.7	0.4
HA Xray ensemble	1.5	1.5	4.0	0.6	0.4
AI Xray ensemble	1.2	1.4	3.8	0.5	0.3
Unrestrained MD ensemble	1.2	1.3	3.2	0.6	0.4
RAM ensemble	0.6	0.7	2.0	0.4	0.2

Table S2, related to Figure 1**.** Chemical shift-based comparison between the ensembles of crystallographic structures of LFA in the LA, IA, HA and AI states, the NOESY-derived structure PDB ID: 1DGQ, the unrestrained MD ensemble and the RAM ensemble. The comparison is made in terms of RMSD (ppm) between the experimentally measured chemical shifts and the values back-calculated from the ensembles using SPARTA+ (Shen and Bax, 2010).

ensemble/chemical shifts	Cα	Cβ	N	H_N	Hα
NMR PDB ID:1DGQ	1.4	1.4	2.7	0.7	0.4
LA Xray ensemble	0.9	1.0	2.2	0.6	0.3
IA Xray ensemble	1.3	1.4	3.2	0.7	0.4
HA Xray ensemble	1.2	1.2	2.7	0.6	0.3
AI Xray ensemble	0.9	1.0	2.2	0.6	0.3
Unrestrained MD ensemble	1.1	1.2	2.8	0.6	0.3
RAM ensemble	0.8	0.9	2.0	0.5	0.2

Supplemental References

Kohlhoff, K.J., Robustelli, P., Cavalli, A., Salvatella, X., and Vendruscolo, M. (2009). Fast and accurate predictions of protein NMR chemical shifts from interatomic distances. J Am Chem Soc *131*, 13894-13895.

Shen, Y., and Bax, A. (2010). Sparta+: A modest improvement in empirical NMR chemical shift prediction by means of an artificial neural network. J Biomol NMR *48*, 13-22.
