# Supplementary material for: Structure and Dynamics of the Integrin LFA-1 I-Domain in the Inactive State Underlie its Inside-Out/Outside-In Signaling and Allosteric Mechanisms
Source: Structure. 2015 Apr 7;23(4):745–53. doi: 10.1016/j.str.2014.12.020 (PMC4396694; doi:10.1016/j.str.2014.12.020)
Supplement: Document S2. Article plus Supplemental Information [file mmc2.pdf]

Structure

Article

# Structure and Dynamics of the Integrin LFA-1 I-Domain in the Inactive State Underlie its Inside-Out/Outside-In Signaling and Allosteric Mechanisms

## Graphical Abstract

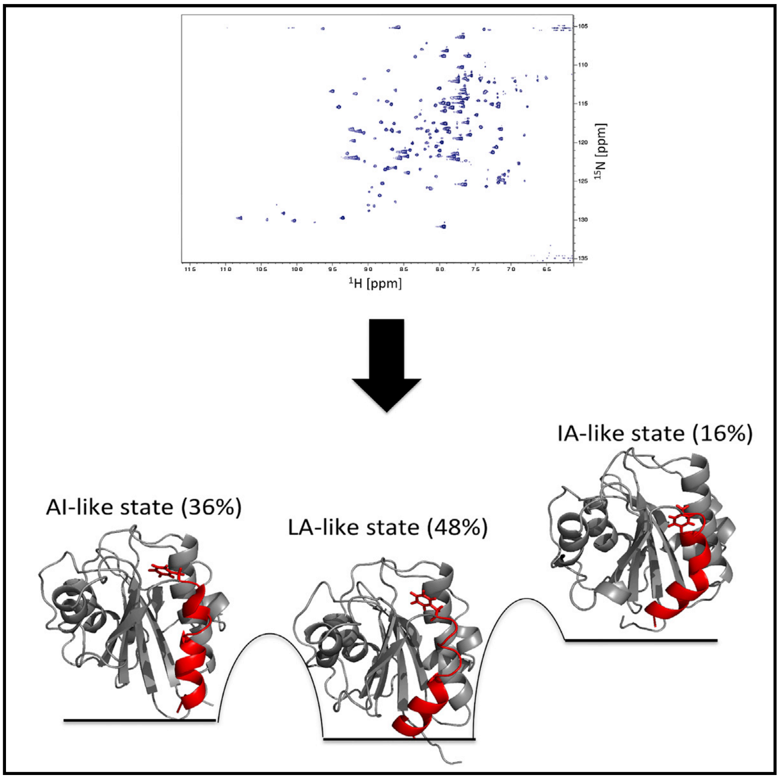


## Highlights

- We identified three substates in the apo LFA-1 I-domain using RAM simulations
- Independent RDC measurements were accurately reproduced using the three substates
- The three substates correspond to the inactive, low, and intermediate affinity states
- The three substates are associated with the precise regulation of the LFA-1 binding

## Authors

Predrag Kukic, Hoi Tik Alvin Leung, ..., Carlo Camilloni, Michele Vendruscolo

## Correspondence

mv245@cam.ac.uk

## In Brief

Kukic et al. identified three substates of the apo LFA-1 I-domain using replica-averaged metadynamics simulations with NMR restraints. The presence of the inactive, low, and intermediate affinity substates in the apo LFA-1 I-domain represents an intrinsic property that can regulate the complex allosteric mechanism of the protein.

Kukic et al., 2015, Structure *23*, 745–753
April 7, 2015 ©2015 The Authors
http://dx.doi.org/10.1016/j.str.2014.12.020

# Structure and Dynamics of the Integrin LFA-1 I-Domain in the Inactive State Underlie its Inside-Out/ Outside-In Signaling and Allosteric Mechanisms

Predrag Kukic,[1] Hoi Tik Alvin Leung,[1,2] Francesco Bemporad,[1,3] Francesco A. Aprile,[1] Janet R. Kumita,[1] Alfonso De Simone,[4] Carlo Camilloni,[1] and Michele Vendruscolo[1,*]
[1]Department of Chemistry, Lensfield Road, University of Cambridge, Cambridge CB2 1EW, UK
[2]Biozentrum, University of Basel, Klingelbergstrasse 50/70, Basel 4056, Switzerland
[3]Dipartimento di Scienze Biomediche Sperimentali e Cliniche, Università degli Studi di Firenze, Viale G. B. Morgagni 50, 50134, Firenze, Italy
[4]Division of Molecular Biosciences, Imperial College London, London SW7 2AZ, UK
*Correspondence: mv245@cam.ac.uk
http://dx.doi.org/10.1016/j.str.2014.12.020
This is an open access article under the CC BY license (http://creativecommons.org/licenses/by/4.0/).

## SUMMARY

**Lymphocyte function-associated antigen 1 (LFA-1) is an integrin that transmits information in two directions across the plasma membrane of leukocytes, in so-called outside-in and inside-out signaling mechanisms. To investigate the structural basis of these mechanisms, we studied the conformational space of the apo I-domain using replica-averaged metadynamics simulations in combination with nuclear magnetic resonance chemical shifts. We thus obtained a free energy landscape that reveals the existence of three conformational substates of this domain. The three substates include conformations similar to existing crystallographic structures of the low-affinity I-domain, the inactive I-domain with an allosteric antagonist inhibitor bound underneath α helix 7, and an intermediate affinity state of the I-domain. The multiple substates were validated with residual dipolar coupling measurements. These results suggest that the presence of three substates in the apo I-domain enables the precise regulation of the binding process that is essential for the physiological function of LFA-1.**

## INTRODUCTION

Integrins are a family of transmembrane receptor proteins that play key roles in mediating the attachment and communication of cells with their environments, including other cells and the extracellular matrix (Barczyk et al., 2010; Hynes, 2002; Ley et al., 2007; Shattil et al., 2010; Takada et al., 2007). They carry out their functions by interacting with cell surface proteins such as cadherins, cell adhesion molecules, selectins, and syndecans, and with extracellular matrix proteins such as collagens, fibronectins, and laminins (Humphries et al., 2006; Hynes, 2009). Lymphocyte function-associated antigen 1 (LFA-1, also known as αLβ2 or CD11a/CD18) is a particularly important integrin exclusively expressed in leukocytes (Kishimoto et al., 1988). This protein regulates the adhesion and migration of leukocytes in the immune response within lymphoid organs, their trafficking at inflammatory sites, and homing within the body (Larson and Springer, 1990; Shimaoka et al., 2002). LFA-1 carries out its function by transmitting information in two directions across the plasma membrane of a leukocyte, through the so-called outside-in and inside-out signaling mechanisms (Dustin and Springer, 1989; Shimaoka et al., 2001). In inside-out signaling, intracellular signals elicited by chemokine and T-cell receptors rapidly upregulate the ability of LFA-1 to bind to its extracellular ligands (Zhang et al., 2009), of which intercellular adhesion molecule 1 (ICAM-1) is of particular biological relevance (Dustin and Springer, 1999). Conversely, during outside-in signaling, the binding of ICAM-1 to LFA-1 triggers the transmission of signals from the extracellular space into the cytoplasm, thereby altering gene expression and cellular metabolism (Luo et al., 2007).

LFA-1 consists of an αL-subunit of 180 kDa and a β2-subunit of 95 kDa. Each subunit is composed of a large N-terminal extracellular domain, a single α-helical transmembrane domain, and a short intracellular domain. Although the extracellular domains of the αL and β2 subunits of LFA-1 are large and structurally complex, the ICAM-1 binding site is contained mainly within the 190-residue Inserted domain (I-domain) of the αL subunit (Shimaoka et al., 2002). The I-domain forms an independent Rossmann-type fold with a central hydrophobic six-stranded β sheet (formed by β strands 1–6) surrounded by seven amphipathic α helices (labeled as α helices 1–7, see Figure S1, related to Figure 1). The ligand binding site is located at the upper face of the I-domain, the so-called metal ion-dependent adhesion site (MIDAS), which coordinates a single $Mg^{2+}$ ion. The distal bottom face of the I-domain bears the N- and C-terminal interface to another domain of the αL subunit, called the β-propeller domain (Xie et al., 2010).

In addition to ICAM-1 binding, the I-domain has been implicated as a critical domain in the outside-in and inside-out signaling of LFA-1 (Shimaoka et al., 2001, 2002, 2003). Outside-in signals are triggered by the I-domain in response to the binding of ICAM-1. In particular, the binding of a negatively charged Glu34 residue within ICAM-1 to the $Mg^{2+}$ ion of MIDAS induces significant structural rearrangements in the loops forming MIDAS, namely the loops between β sheet 1 and α helix 1, α helix 3 and α helix 4, and β sheet 4 and α helix 5 (Shimaoka et al., 2003). The rearrangements of MIDAS in this ligand bound open conformation

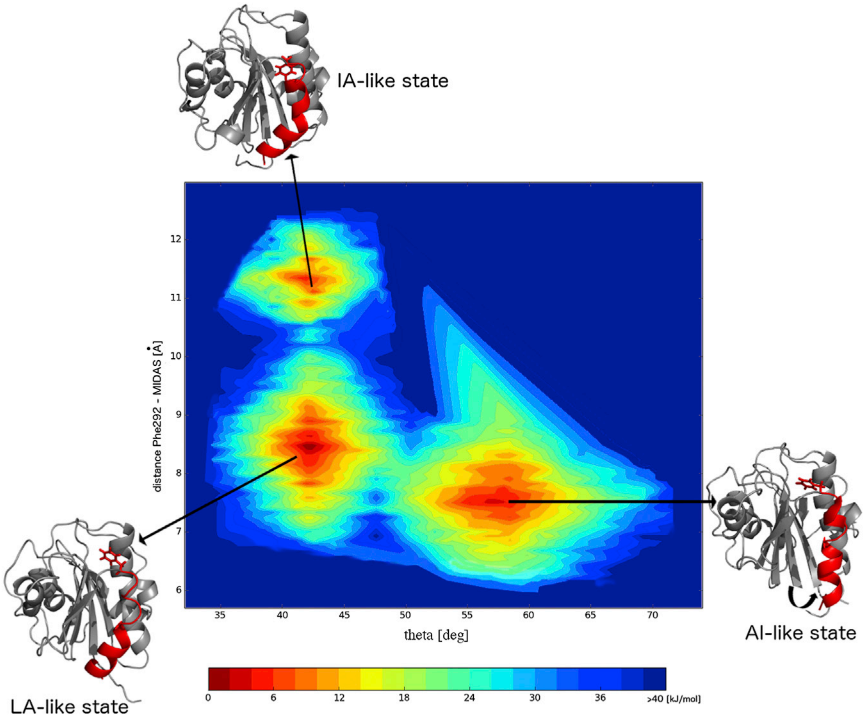


**Figure 1. Three-Dimensional Representation of the Free Energy Landscape of the LFA-1 I-Domain**

The free energy landscape is represented as a function of the angle θ that α helix 7 forms with the hydrophobic core of the I-domain and the distance between the side chain of Phe292 and the $Mg^{2+}$ ion in MIDAS (see Figure S1B, related to Figure 1). α helix 7 and the side chain of Phe292 are shown in red. Two lowest energy minima represent the LA-like and AI-like states, whereas the highest energy minimum represents the IA-like state. deg, degrees.

cause a large 10-Å movement of the C-terminal α helix 7 down the side of the I-domain. This disposition of α helix 7 further induces global structural rearrangements in other domains of LFA-1 and finally its activation (Campbell and Humphries, 2011).

The inactive I-domain is normally maintained in the so-called closed conformation, which has a low affinity (LA) for ICAM-1. However, during the inside-out signaling, intracellular signals received by the LFA-1 cytoplasmic domains make the I-domain much more competent for ligand binding. The conformational changes from the cytoplasmic domains are further transferred to the β I-like domain of the αL subunit. The β I-like domain MIDAS has been proposed to bind Glu310, located in the linker following α helix 7, and exerts a pull on α helix 7 that further induces the rearrangements at the I-domain MIDAS (Lu et al., 2001a). So far, this mechanism of activation has been supported by mutagenesis studies that were able to stabilize intermediate affinity (IA) and high affinity (HA) states of the I-domain without the presence of ligands (Shimaoka et al., 2001, 2003). In those studies, disulfide bonds introduced by mutagenesis were engineered to connect α helix 7 with its neighboring β strand 6 or α helix 1. Such disulfide bonds were able to pull α helix 7 down the side of the I-domain and induce a conformational change at MIDAS. In the HA state, the conformations of both MIDAS and α helix 7 were very similar to the open ligated conformation and showed an increase in the affinity for ICAM-1 by 10,000-fold (Shimaoka et al., 2001). In the IA state, the position of α helix 7 was engineered to be halfway down the side of the I-domain. Although this disposition of α helix 7 in the IA state kept MIDAS in the close conformation, the IA state showed an increase in the affinity for ICAM-1 by 500-fold compared with its affinity in the LA state (Shimaoka et al., 2003). In both IA and HA states, residue Phe292, which is positioned on the top of α helix 7 and deeply buried in the hydrophobic pocket (so-called ratchet pocket) was displaced from the hydrophobic pocket (see Figure S1B, related to Figure 1). The displacement of Phe292 from the ratchet pocket is crucial in unlocking α helix 7 and enables its movement along the side of the I-domain.

Both the inside-out and the outside-in signaling mechanisms can be inhibited by the binding of the I-domain inhibitors to the pocket underneath α helix 7 (Last-Barney et al., 2001; Weitz-Schmidt et al., 2001), which results in an allosterically inhibited (AI) state. These inhibitors, termed α-I allosteric antagonists, bind and stabilize the closed state of the I-domain and inhibit its conversion to the HA state. Their mode of action involves blocking of the downward axial displacement of α helix 7, which in turn allosterically inhibits ICAM-1 binding to the distal MIDAS site. This mode of action was confirmed by a mutant I-domain that was locked in the HA state with an engineered disulphide bond. LFA-1 with the I-domain locked in the HA open conformation showed resistance to inhibition by the α-I allosteric antagonists (Lu et al., 2001b).

The structure-affinity relationship of the I-domain led to the suggestion that its affinity is regulated by its conformation and that the allosteric link between MIDAS and the motion of α helix 7 is critical in the binding regulation and the transmission of the outside-in and inside-out signals. Although the general mechanism that governs this conformational dependence of affinity has been laid out by structural and biophysical studies, a complete understanding of the intrinsic dynamics of the LFA-1 I-domain is currently lacking. To investigate this problem, we set out to characterize the dynamics of the I-domain in the apo form and the way it facilitates the complex signaling and allosteric modes of LFA-1.

In a previous study, the apo I-domain dynamics were studied on a broad range of timescales (Leung et al., 2014). Short timescale dynamics, determined from $R_1$ and $R_2$ relaxation rates and the model-free analysis, showed very restricted backbone motions on the pico- to nanosecond timescales. Surprisingly, almost all reported $S^2$ values of the backbone amides were above 0.9. On the contrary, residual dipolar coupling (RDC) measurements from the same study unequivocally showed large discrepancies with all crystallographic structures determined to date, which were particularly large for the residues that belong to α helix 7. These results led to a proposal that α helix 7 is in

dynamic exchange between many conformations on the microsecond to millisecond timescales and that the motion of $\alpha$ helix 7 in the apo I-domain can be crucial for the function of LFA-1.

In this study, we obtained a conformational ensemble that represents the dynamics of the apo I-domain and illustrates the way these dynamics facilitate the complex signaling and allosteric modes of LFA-1. In particular, the low-frequency motions of the apo I-domain were characterized with a recently developed replica-averaged metadynamics (RAM) methodology. The RAM methodology integrates experimentally measured nuclear magnetic resonance (NMR) chemical shifts with bias exchange metadynamics, which is an advanced sampling method particularly effective in exploring the conformational space of proteins. The NMR chemical shifts help decode the chemical environment of atomic nuclei on timescales similar to those obtained from RDC measurements, and the RAM simulations are able to explore low-probability regions of the free energy landscape by allowing free diffusion along particular reaction coordinates (or collective variables [CVs]). The results obtained from this work strongly support the idea that not only the LA state but also the AI and IA states are embedded in the dynamics of the apo form of the I-domain. The existence of multiple states was then further validated with the previously measured RDCs. The presence of the multiple affinity states in the apo I-domain allows more precise regulation of ligand binding and is most probably essential for the physiological function of LFA-1.

## RESULTS

### Choice of the CVs

Experimentally measured backbone chemical shifts of LFA-1 (Kriwacki et al., 2000) were used as replica-averaged restraints in the RAM simulations (Camilloni et al., 2013a, 2013b) (see the Experimental Procedures section). The use of RAM methodology in studying the dynamics of LFA-1 has two major advantages in comparison with the use of conventional molecular dynamics simulations. The first is that this method exploits effectively the ability of NMR chemical shift restraints to generate an ensemble of conformations compatible with the given set of experimental measurements in the sense of the maximum entropy principle (Cavalli et al., 2013; Pitera and Chodera, 2012; Roux and Weare, 2013). In this way, the use of chemical shifts has enabled the characterization of the interdomain motions of ribonuclease A (Camilloni et al., 2012a) and calmodulin (Kukic et al., 2014). The second advantage is the fact that the RAM approach makes it possible to explore low-probability regions of the free energy surface of LFA-1 and at the same time allows its free diffusion along the CVs of LFA-1.

For each replica in the RAM simulations, we use a different metadynamics history-dependent potential (Laio and Parrinello, 2002) acting on a different CV (see the Experimental Procedures section). Three CVs defined here act at the tertiary structure level by biasing the dihedral angles of the I-domain. The first CV acts on the $\phi$ and $\varphi$ dihedral angles of all residues in the protein; the second CV acts on the $\chi1$ angles of all heavy side chain residues (Phe, His, Tyr, Trp, Arg, Lys, and Met) except the residues that belong to $\alpha$ helices 1 and 7; the third CV acts on $\chi1$ angles of the heavy side chain residues that belong solely to $\alpha$ helices 1 and 7. Hydrophobic interactions between $\alpha$ helices 1 and 7 have been given particular importance in the allosteric coupling between $\alpha$ helix 7 and MIDAS in previous studies (Gaillard et al., 2007; Shimaoka et al., 2003), and hence, these interactions were encoded solely by the third CV. Lastly, the fourth CV biases the angle $\theta$ that $\alpha$ helix 7 forms with the hydrophobic core of the I-domain (Figure S1B, related to Figure 1).

### Thermodynamics of the apo LFA-1 I-Domain

The RAM simulations of the LFA-1 I-domain reached convergence after 150 ns when the bias potentials acting on all four replicas started to become stationary (Marinelli et al., 2009). The simulations were then further run for an additional 80 ns to reconstruct the free energy landscape of the I-domain. In Figure 1, this free energy landscape is represented as a function of the angle $\theta$ that $\alpha$ helix 7 forms with the hydrophobic core of the I-domain and the distance between the Phe292 side chain and the $Mg^{2+}$ of MIDAS.

The free energy landscape clearly reveals the existence of three distinct minima (Figure 1). The two lowest minima differ by just 0.7 kJ/mol, a value well within the thermal fluctuations and comparable with the error of the WHAM algorithm (Marinelli et al., 2009) used to recover the free energy surface (see the Experimental Procedures section). These two minima can be distinguished by different values of the angle $\theta$ that $\alpha$ helix 7 forms with the hydrophobic core of the I-domain. The lowest minimum includes conformations in which $\alpha$ helix 7 is bound to the hydrophobic core of the I-domain with angle $\theta$ visiting values similar to those found in the crystallographic structures of the apo I-domain in the LA state (Figure 2A). Therefore, from here on, this state is referred to as the LA-like state. The second free energy minimum includes conformations in which $\alpha$ helix 7 swings out of the I-domain with the angle $\theta$ visiting a range of values similar to those found in I-domain structures with the $\alpha$-I allosteric antagonists bound underneath the $\alpha$ helix 7, i.e. the AI state (Figure 2A). Hence, this state is referred to as the AI-like state. Both the LA-like and AI-like states have the side chain of Phe292 deeply buried inside the ratchet pocket.

Unrestrained simulations, unlike the chemical shift restrained RAM simulations, were only able to sample values of the angle $\theta$ similar to those obtained in the LA-like state (Figure 2B). On the contrary, the only solution structure of the apo LFA-1 I-domain solved by the conventional nuclear Overhauser effect spectroscopy (NOESY)-based approach (Legge et al., 2000) samples the values of the angle $\theta$ in a range similar to those found in the AI-like state (Figure 2B). Both the unrestrained ensemble and NOESY-derived ensemble have the side chain of Phe292 deeply buried inside the ratchet pocket.

The conformations identified in the highest energy minimum can be separated from the rest of the structures in the RAM ensemble by examining the distance between the side chain of residue Phe292 and the MIDAS site (Figure 3B). Whereas this distance in the LA-like and AI-like conformations has an average value of 8 Å, the conformations in the highest free energy minimum have an average value considerably larger, i.e. 11.8 Å. Because the side chain of Phe292 is displaced from the ratchet pocket in the highest energy minimum, we will refer to this state as the IA-like state. However, the IA-like conformations identified here differ from the IA state obtained from the mutagenesis studies (Shimaoka et al., 2001, 2003). In the IA state, the

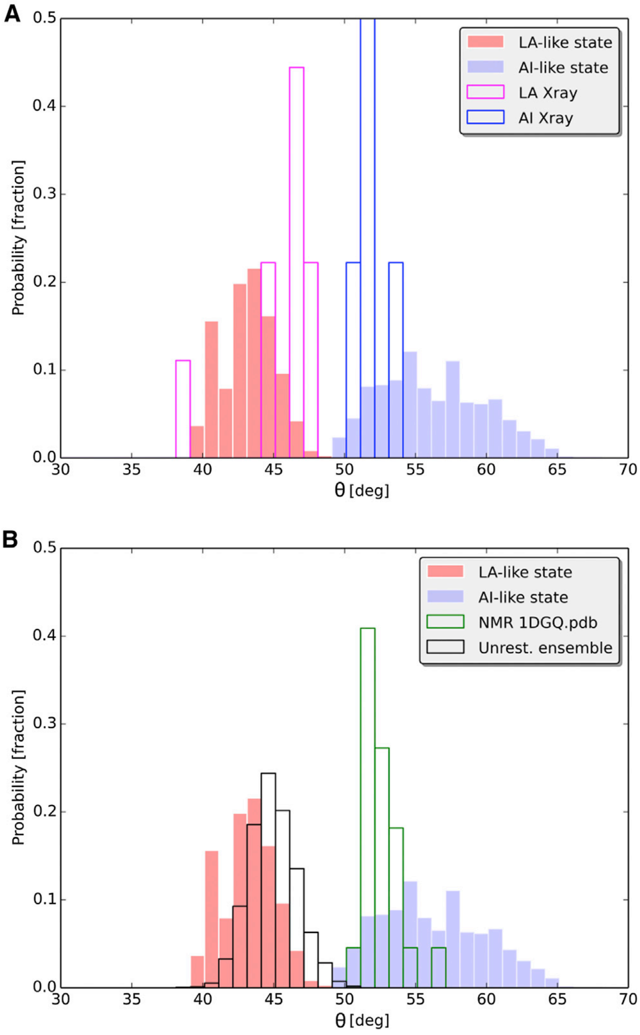


**Figure 2. Characterization of the Breathing Motion of α Helix 7**

(A and B) The distribution of the θ angle from the RAM ensemble is represented by filled bars (red, LA-like state; blue, AI-like state). This distribution is compared with that obtained from (A) the ensemble of X-ray structures in the LA (red bars) and AI states (blue bars) and (B) the unrestrained (Unrest.) ensemble (black bars) and the NMR structure PDB ID 1DGQ (green bars). deg, degrees.

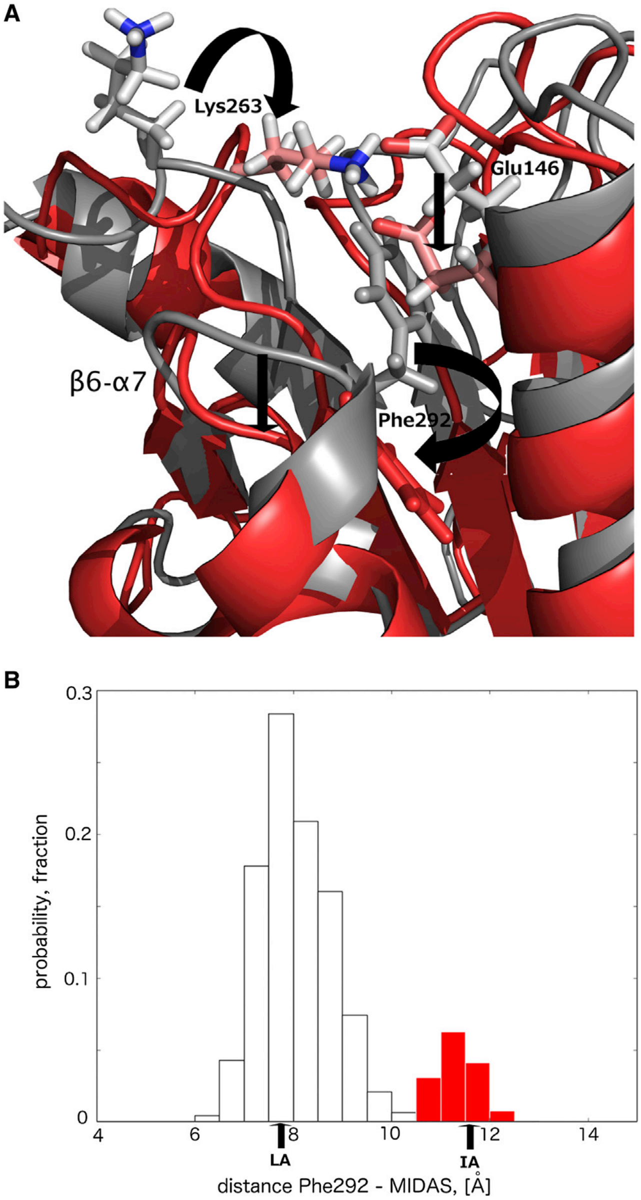


**Figure 3. Analysis of the Structural Properties of the IA-like State**

(A) The movement of the side chain of Phe292 outside the ratchet pocket induces formation of a salt bridge between residues Glu146 and Lys263, and depression of the loop between β sheet 6 and α helix 7 down the side of the I-domain in the IA-like state from the RAM ensemble (red). The LA-like state from the RAM ensemble is depicted in gray.

(B) Distance distribution between the Cz atom of residue Phe292 and the $Mg^{2+}$ ion in the IA-like state from the RAM ensemble (red bars). The same distance distribution in the LA-like and AI-like states from the RAM ensemble is shown in black outlined bars. Corresponding distances from the LA and IA crystallographic structures are depicted as references by the arrows at the bottom of the figure.

introduced disulfide bond L161C-F299C pulls α helix 7 down the side of the I-domain for a single α-helical turn. As a consequence, residue Phe292, which occupies the deeply buried hydrophobic pocket in the LA state, is replaced by residue Leu289 in the ratchet position (Shimaoka et al., 2002). On the other hand, the IA-like conformations observed in the RAM ensemble have residue Phe292 outside the hydrophobic pocket. However, the displacement is not significant enough to bring Leu289 into the ratchet position. The displacement of the side chain of Phe292 from the ratchet position in the IA-like conformations, however, closely mimics an early stage necessary for the movement of α helix 7 down the side of the I-domain in the IA state identified by the mutagenesis results. As a consequence, the loop between β sheet 6 and α helix 7 that connects α helix 7 with the rest of the protein is displaced in the direction of the IA and HA states and differs markedly from its conformation in the LA state (Figure 3B).

Displacement of the side chain of residue Phe292 out of the hydrophobic pocket in the IA-like state is followed by the formation of the salt bridge between Glu146 and Lys263 (Figure 3B),

which causes an inward rigid body movement of the α helix 1 that needs now to shield the hydrophobic core from the solvent. This motion of α helix 1 is essentially identical to that observed in the transition from the LA to the HA states of LFA-1 (Shimaoka et al., 2001, 2002, 2003). The motion of the α helix 1 causes rearrangements in β strand 6, which connects α helix 7 with the rest of the protein, and further rearrangements of hydrophobic interactions between β strand 6 and α helix 6. As a consequence, α helix 6 appears to be displaced in the same direction as during the transition from the LA to the HA states of LFA-1 (see Figure S4A, related to Figure 3, for the superposition of the LA-like and IA-like states and Figure S4B, related to Figure 3, for the superposition of the LA and HA states).

The conformational rearrangements in the IA-like state do not cause significant backbone conformational changes elsewhere in the I-domain and hence leave MIDAS in the closed conformation, in accordance with the conclusions of the mutagenesis studies (Shimaoka et al., 2001, 2003). However, the IA-like state is now primed for further structural rearrangements, similar to those that appear during the transition from the LA to the IA/HA state. The key residue Phe292, in the loop between β sheet 6 and α helix 7, is already removed from its hydrophobic pocket and the loop is moved away from the hydrophobic core, whereas α helix 1 is displaced closer to the hydrophobic core and α helix 6 in the direction of the HA state. Therefore, the backbone flip of the loop between β sheet 4 and α helix 5 at residue Gly240, which was previously identified as necessary for the opening of MIDAS in the HA state (Shimaoka et al., 2001, 2002, 2003), is now achievable with a significantly lower energetic cost in comparison with the LA state.

The conformations from the IA-like state appear in the free energy landscape with a free energy of at least 2.8 kJ/mol higher than that of the global minimum of the LA-like state. From these relative free energies, we can calculate the populations of the three states at 300 K, which are 48% for the LA-like state, 36% for the AI-like state, and 16% for IA-like states.

## Convergence of the RAM Simulations

The convergence of the RAM simulations was confirmed by the comparison between the experimentally measured chemical shift values and the values back-calculated from the RAM ensemble (Tables S1 and S2, related to Figure 1, and Figures S2 and S3, related to Figure 1). The chemical shifts were back-calculated using two methods, the CamShift (Kohlhoff et al., 2009) and the Sparta+ methods (Shen and Bax, 2010), whereby the latter was not employed to calculate the restraints during the simulations. The ensemble-averaged chemical shifts obtained from the RAM ensemble are in agreement with those measured experimentally (Tables S1 and S2, related to Figure 1, and Figures S2 and S3, related to Figure 1). This was not the case when the chemical shifts were predicted from the deposited X-ray and NMR structures of the I-domain found in the LA, IA, HA, and AI states (Tables S1 and S2, related to Figure 1, and Figures S2 and S3, related to Figure 1). Moreover, the back-calculated values from the RAM ensemble were in better agreement with the experiment than the corresponding values back-calculated from the unrestrained ensemble. These results illustrate the importance of including experimental observables as restraints in molecular dynamics simulations.

The convergence of the RAM ensemble was further assessed by calculating the secondary structure populations of the I-domain (Figure 4). As chemical shifts can provide accurate information about these populations, we first performed the calculations for the I-domain from experimental chemical shifts (Camilloni et al., 2012a). The experimentally determined secondary structure populations were then compared with those back-calculated from the RAM and unrestrained ensemble using the DSSP program (Kabsch and Sander, 2004). The results show that the secondary structure populations in the RAM ensemble are in close agreement with the experimentally derived ones (Figure 4A, root-mean-square deviation $[rmsd]_{\alpha\ helix} = 0.18$, $rmsd_{\beta\ sheet} = 0.19$). This was not the case when the secondary structure populations were obtained from the unrestrained ensemble (Figure 4B, $rmsd_{\alpha\ helix} = 0.27$, $rmsd_{\beta\ sheet} = 0.26$). In particular, the populations of α helices 5, 6, and 7 were not accurately reproduced. Most importantly, the RAM ensemble was able to reproduce the complex secondary structure composition of α helix 7 derived from experimentally measured chemical shifts: the N-terminal residues (292–295) in the $3_{10}$ conformation similar to the conformation observed in the crystallographic structures (Shimaoka et al., 2003), the central residues (296–298) mainly lacking any secondary structure, and the C-terminal residues (299–304) in the typical α-helical conformation. The complex secondary structure distribution along the α helix 7 residues is most probably intimately related to the highly dynamic nature of this α helix and explains the existence of multiple states in the apo form of the I-domain.

## Validation with RDC Measurements

A previous study reported the measurement of 135 $^{15}N$-$^{1}H$ RDCs of the I-domain in filamentous bacteriophages Pf1 media (Leung et al., 2014). In the same study, the level of agreement between the RDC values and the existing crystallographic and NMR structures of the LFA-1 I-domain was assessed using the Q factor. The magnitude and orientation of the alignment tensor relative to the molecular frame were obtained in this study using a singular value decomposition-based method (Losonczi et al., 1999), in which the RDCs of residues belonging to β strands 1, 2, 4, and 5 were used for the calculation of alignment tensors. These β strands belong to the central β sheet and correspond to the region with the lowest crystallographic B factor in the protein (Leung et al., 2014). The RDCs of the remaining residues were back-calculated and used to determine the Q factors. The lowest value of the Q factor was reported for the ensemble of PDB structures that represent the AI (Q factor 0.4) and LA state (Q factor 0.46). Interestingly, almost equal populations of these two states were identified here in the apo I-domain using the RAM approach. Higher Q factors were reported for structures in the IA (Q factor 0.58) and HA states (Q factor 0.58), whereas the structures of the apo I-domain obtained from the NOESY restraints reproduced the RDC values with the highest Q factor (Q factor 0.76). Regardless of the activation state of the I-domain, the Q factors corresponding to α helix 7 were consistently highest among all secondary structure elements in the protein. The upper and lower limits of the α helix 7 Q factors were 0.8 reported for the AI state ensemble and 1.53 reported for the NOESY-derived structures.

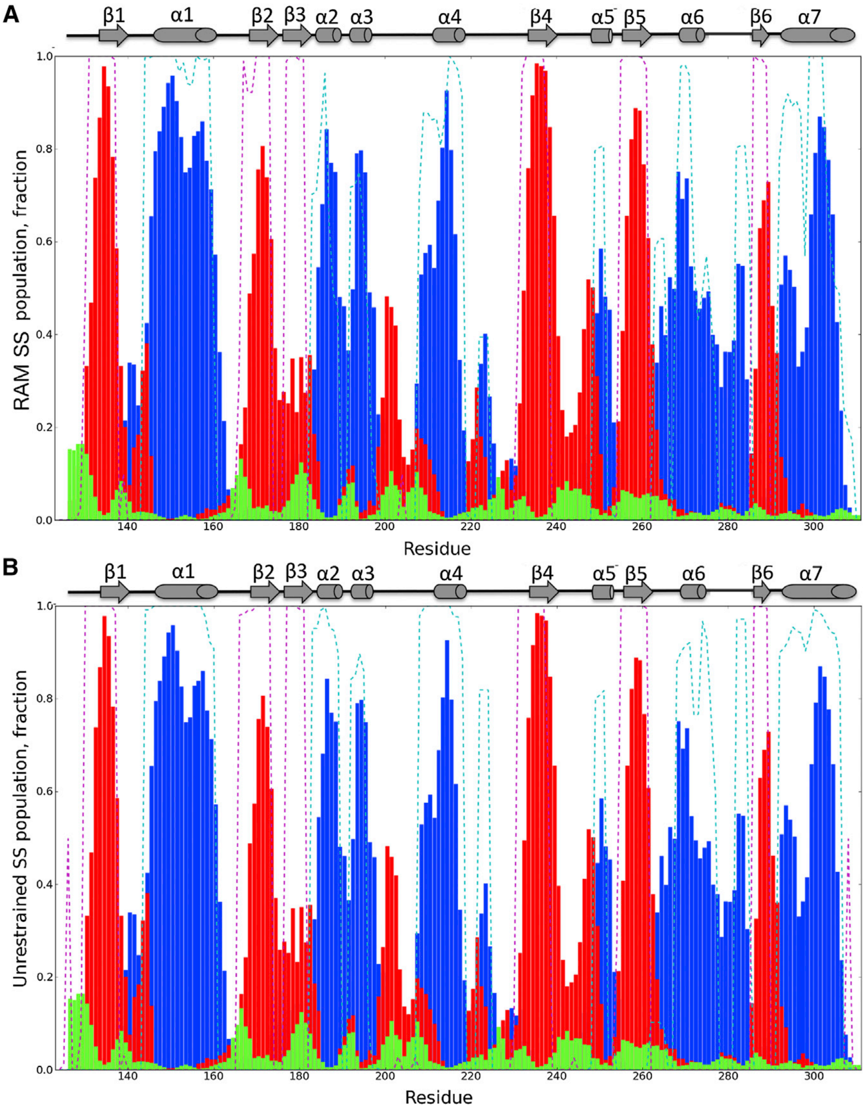


**Figure 4. Secondary Structure Populations of the I-Domain**

(A and B) We compared the populations derived for the δ2D method (Camilloni et al., 2012a) and experimental chemical shifts (filled bars) and obtained from the RAM ensemble (A, dashed line), and the unrestrained molecular dynamics ensemble (B, dashed line). Blue, red, and green indicate α helix, β sheet, and polyproline II populations, respectively.

Here, the measured RDC values are also used as criteria for the validity of the RAM ensemble and the ensemble generated from the unrestrained simulations (Figure 5). The Q factor obtained from the RAM ensemble was 0.36, whereas the corresponding value for the unrestrained ensemble was 0.55. The RAM ensemble showed better agreement with the RDC values than any other ensemble of the PDB structures reported previously or any other individual crystallographic structure (Leung et al., 2014) (Figure 5). Furthermore, the RAM ensemble improved the agreement with the RDC measurements for all secondary structure elements in the I-domain in comparison with the individual crystallographic structures and their ensembles in different states, except for the MIDAS site, where the agreement was in the same range. Interestingly, the largest improvement in the Q factor was obtained for α helix 7, with a value of 0.28 obtained from the RAM ensemble. This value was more than 0.5 lower than the corresponding values obtained from the ensembles of crystallography structures in different states. The corresponding Q factor for α helix 7 obtained from the unrestrained ensemble was still high (0.61). The reason for this significant improvement in the Q factor in the RAM ensemble most probably comes from its ability to reproduce the complex secondary structure population along the α helix 7 residues and the lack of this ability in the unrestrained ensemble (Figure 4).

## DISCUSSION

In this study, we have characterized the intrinsic flexibility of the apo I-domain, thereby identifying three substates, which resemble those populated during the function of the protein (Figure 6). The first substate includes conformations similar to the existing crystallographic structures of the LA I-domain (LA-like state), the second substate conformations that resemble the crystallographic structures of the I-domain with an allosteric antagonist bound underneath α helix 7 (AI-like state), and the third substate conformations similar to the IA state of the I-domain (IA-like state). These three substates mainly differ in the conformation of α helix 7 and in the loop that anchors α helix 7 to β sheet 6 and therefore to the rest of the domain. These results are in good agreement with previous experimental measurements (Shimaoka et al., 2001, 2003) and a normal mode analysis-based study (Gaillard et al., 2007) that identified α helix 7 and the loop between α helix 7 and β sheet 6 as being critically important for the bidirectional transmission of biochemical and mechanical signals of LFA-1 across the plasma membrane. Moreover, the RAM ensemble identified here is in considerably better agreement with the previous RDC measurements than any existing crystallographic structure of the I-domain or ensemble formed from these structures.

In addition to the conventional position of α helix 7 found in the LA state, the apo I-domain also encodes the positions of α helix 7 similar to those found in the AI and IA states. The main characteristic of the α helix 7 in the AI-like state identified here is its swing-out displacement anchored at the ratchet pocket. This breathing motion of α helix 7 is able to sufficiently increase the volume of the allosteric pocket in the apo I-domain. This finding is consistent with the limited protection from deuterium

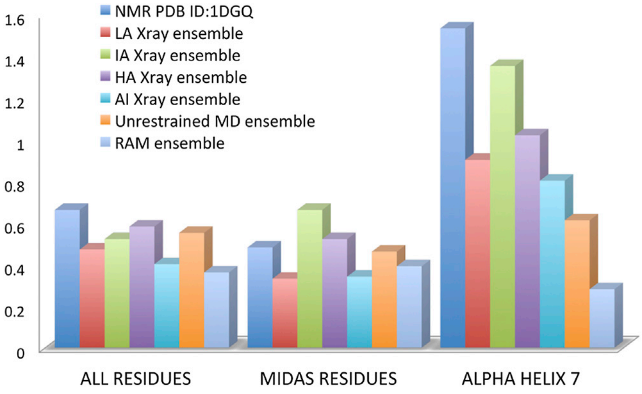


**Figure 5. Validation of the RAM Ensemble Using RDCs**

The $^{15}N$-$^{1}H$ RDCs were measured with *Pf1* bacteriophage at 0 mM NaCl concentration. The LA, AI, IA, and HA states are formed as ensembles of available crystallographic structures in corresponding states present in the PDB.

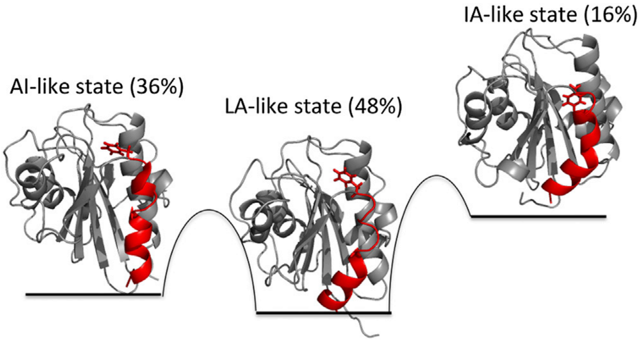


**Figure 6. Schematic Illustration of the Three Major Substates in the Free Energy Landscape of the I-Domain in the apo State**

The first substate includes conformations similar to existing crystallographic structures of the LA I-domain (LA-like state), the second substate conformations that resemble crystallographic structures of the I-domain with an allosteric antagonist bound underneath α helix 7 (AI-like state), and the third substate conformations similar to the IA state of the I-domain (IA-like state).

exchange in α helix 7 measured for the apo I-domain and with the low density of long-range nuclear Overhauser effects that α helix 7 residues form with the rest of the protein (Legge et al., 2000). Furthermore, the RDC analysis shows that this swing-out conformation of α helix 7 is as probable as the bound conformation typical for the LA state. Therefore, the AI-like state of the apo I-domain appears to be an intrinsic property of the I-domain and is most probably relevant for controlling access to the allosteric binding pocket.

The main characteristic of the IA-like state identified here is the displacement of the side chain of residue Phe292 from the hydrophobic ratchet pocket and subsequent displacement of α helix 7 and the loop between α helix 7 and β sheet 6. The crucial role of residue Phe292 in the allosteric mechanism of LFA-1 has previously been identified (Jin et al., 2006). The substitution of this residue with Ala and Gly has been shown to increase the binding affinity for ICAM-1 by 75-fold and 1300-fold, respectively, in comparison with the wild-type protein (Jin et al., 2006). Moreover, the Phe292Ala and Phe292Gly mutants experienced 3-fold and 5-fold higher association rates to ICAM-1 than the wild-type. The motion of α helix 7 in the IA-like state identified here is not of the same amplitude as the piston-like motion identified by previous mutagenesis studies (Shimaoka et al., 2001, 2003). However, α helix 7 appears to be displaced in the same direction as during the transition from the LA to the IA and HA states and resembles the early stages of this transition. This IA-like state appears to be an intrinsic property of the apo I-domain and may play a crucial role in LFA-1 activation and the transmission of signals in both directions. These results suggest that the presence of multiple substates in the apo I-domain, which are likely to have different ligand affinities, allows a precise regulation of ligand binding and is most probably essential for the physiological function of LFA-1.

## EXPERIMENTAL PROCEDURES

The simulations described here were performed using the Amber ff99SB* force field (Best and Hummer, 2009) with the TIP3P water model (Jorgensen et al., 1983). They were run using the GROMACS computational suite (Hess et al., 2008) modified with PLUMED (Tribello et al., 2014) and Almost (Cavalli et al., 2005; Fu et al., 2014). The starting conformation was taken from a crystallography-obtained structure (PDB ID, 1LFA). This structure was initially solvated in a water box extending 12 Å from its surface and then energy minimized. All simulations were carried out in the canonical ensemble by keeping the volume fixed and by thermosetting the system with the modified Berendsen thermostat (Berendsen et al., 1984). The net charge was neutralized by adding two $Na^+$ ions. The system was evolved with a time step of 2 fs by constraining the fast-bonded modes using LINCS (Hess et al., 2008). van der Waals interactions were accounted for by using a cut-off of 12 Å. The particle mesh Ewald method (Essmann et al., 1995) with a grid spacing of 1.09 Å was used for the electrostatic contribution to nonbonded interactions. The RAM ensemble was obtained using chemical shift restraints and RAM sampling, as explained below.

### Chemical Shift Restraints

The force field was modified using a chemical shift-based energy term defined as (Camilloni et al., 2012b)

$$E_{CS} = \sum_{i=1}^{183} \sum_{j=1}^{6} E_{ij}\left(\delta_{ij}^{calc} - \delta_{ij}^{exp}\right), \qquad \text{(Equation 1)}$$

where $E_{ij}$ is a chemical shift-based energy term corresponding to an atom of type $j$ (e.g. Hα, HN, N, Cα, Cβ, C′) and to the $i$th residue in the protein (Kohlhoff et al., 2009; Robustelli et al., 2010). The experimental chemical shifts are denoted by $\delta_{ij}^{exp}$, and their corresponding calculated values $\delta_{ij}^{calc}$ are obtained as averages over four replicas (Camilloni et al., 2012b). The inclusion of replica-averaged chemical shifts into the force field generated an ensemble of the LFA-1 I-domain compatible with the given set of NMR chemical shifts in the sense of the maximum entropy principle (Cavalli et al., 2013; Pitera and Chodera, 2012; Roux and Weare, 2013).

### The RAM Ensemble

The RAM simulations (Camilloni et al., 2013b; Camilloni and Vendruscolo, 2014; Piana and Laio, 2007) were carried out by combining two advanced sampling methods, replica exchange (Sugita and Okamoto, 1999) and metadynamics (Laio and Parrinello, 2002). The first, replica exchange, is particularly effective in overcoming the multiple minima problem on a rugged energy surface through the exchange of conformations between multiple replicas (Sugita and Okamoto, 1999). The second method, metadynamics, is an algorithm that efficiently computes free energies and explores the reaction pathways (Laio and Parrinello, 2002). It is based on a dynamics performed in the space of specific functions of the atomic coordinates (CVs) (Laio and Parrinello, 2002). The

dynamics are driven by a free energy biased by a time-dependent Gaussian potential centered along the trajectory of the CVs:

$$V(\vec{s},t)=\sum_{k\tau<t}W(k\tau)\exp\left(-\sum_{i=1}^{d}\frac{\left(s_i-s_i^{(0)}(k\tau)\right)^2}{2\sigma_i^2}\right). \quad \text{(Equation 2)}$$

In this equation, $d$ is the number of CVs used, $\tau$ is the time step at which the contributions to the bias potential are added, $s^{(0)}$ specifies the position of the centroid of the Gaussian, and $W$ and $\sigma_i$ are the Gaussian height and width, respectively. The history-dependent bias potential defined by Equation 2 discourages the simulations from exploring regions already visited and therefore significantly enhances the sampling speed.

### Choice of the CVs

We performed RAM simulations of the LFA-1 I-domain at 300 K, using four replicas, one for each of the following four CVs:

CV1: dihedral correlation. CV1 acts on 374 $\phi$ and $\varphi$ dihedral angles of all residues in the protein. Parameters: Gaussian width $\sigma$ = 0.1.

CV2: dihedral correlation. CV2 acts on 35 $\chi$1 angles that belong to all heavy side chain residues (Phe, His, Tyr, Trp, Arg, Lys, and Met) except for residues in $\alpha$ helices 1 and 7. Parameters: Gaussian width $\sigma$ = 0.1.

CV3: dihedral correlation. CV3 acts on 25 $\chi$1 angles that belong to the heavy side chain residues of $\alpha$ helices 1 and 7. Parameters: Gaussian width $\sigma$ = 0.1.

CV4: angle. CV4 uses the angle $\theta$ that $\alpha$ helix 7 forms with the hydrophobic core of the I-domain (Figure S1B, related to Figure 1). The $\theta$ angle is defined by the centers of mass of three groups of C$\alpha$ atoms (Arg256-Gly260, Asp290-Phe292, Leu305-Ile316). Parameters: Gaussian width $\sigma$ = 0.05.

### Free Energy Reconstruction in the CV Space

The bias potentials became stable after $t_{eq}$~150 ns (Marinelli et al., 2009). The simulation was further run for an additional 80 ns to reconstruct the free energy landscape of the protein. All the analyses were performed as previously described (Marinelli et al., 2009) using METAGUI (Biarnes et al., 2012) and a visual molecular dynamics (Humphrey et al., 1996) interface for analyzing metadynamics and molecular dynamics simulations. In total, 4885 microstates were identified and their free energy values were computed according to the corresponding bias potentials and the populations observed after the $t_{eq}$.

### The Unrestrained Molecular Dynamics Ensemble

To investigate the effects of the chemical shift restraints on the results, we repeated the simulations with the same protocol used for the RAM ensemble, but without the chemical shift restraints.

### The PDB Ensemble

In addition to the RAM and unrestrained ensembles, we created ensembles of the LFA-1 I-domain from the structures available in PDB. This ensemble includes structures of the I-domain in the LA state (PDB ID, 1LFA, 3F74, 1ZOO, 1ZOP), IA state (PDB ID, 1MJN, 1MQ8), HA state (PDB ID, 1MQA, 1MQ9, 3EOA, 3EOB, 3HI6, 3BN3, 3TCX), and AI state (PDB ID, 3F78, 2O7N, 2ICA, 3M6F, 1XDD, 1XDG, 3BQM, 3BQN, 1CQP, 3E2M, 1XUO, 1RD4).

## SUPPLEMENTAL INFORMATION

Supplemental Information includes four figures and two tables and can be found with this article online at http://dx.doi.org/10.1016/j.str.2014.12.020.

## AUTHOR CONTRIBUTIONS

All authors participated in the research. All authors participated in writing and editing the final manuscript.

## ACKNOWLEDGMENTS

This study was supported by the Wellcome Trust and the BBSRC.

Received: October 15, 2014
Revised: December 4, 2014
Accepted: December 6, 2014
Published: March 12, 2015

## REFERENCES

Barczyk, M., Carracedo, S., and Gullberg, D. (2010). Integrins. Cell Tissue Res. *339*, 269–280.

Berendsen, H.J., Postma, J.P.M., van Gunsteren, W.F., DiNola, A., and Haak, J. (1984). Molecular dynamics with coupling to an external bath. J. Chem. Phys. *81*, 3684–3690.

Best, R., and Hummer, G. (2009). Optimized molecular dynamics force fields applied to the helix-coil transition of polypeptides. J. Phys. Chem. B *113*, 9004–9015.

Biarnes, X., Pietrucci, F., Marinelli, F., and Laio, A. (2012). METAGUI. A VMD interface for analyzing metadynamics and molecular dynamics simulations. Comput. Phys. Commun. *183*, 203–211.

Camilloni, C., and Vendruscolo, M. (2014). Statistical mechanics of the denatured state of a protein using replica-averaged metadynamics. J. Am. Chem. Soc. *136*, 8982–8991.

Camilloni, C., De Simone, A., Vranken, W., and Vendruscolo, M. (2012a). Determination of secondary structure populations in disordered states of proteins using nuclear magnetic resonance chemical shifts. Biochemistry *51*, 2224–2231.

Camilloni, C., Robustelli, P., Simone, A.D., Cavalli, A., and Vendruscolo, M. (2012b). Characterization of the conformational equilibrium between the two major substates of RNase A using NMR chemical shifts. J. Am. Chem. Soc. *134*, 3968–3971.

Camilloni, C., Cavalli, A., and Vendruscolo, M. (2013a). Assessment of the use of NMR chemical shifts as replica-averaged structural restraints in molecular dynamics simulations to characterize the dynamics of proteins. J. Phys. Chem. B *117*, 1838–1843.

Camilloni, C., Cavalli, A., and Vendruscolo, M. (2013b). Replica-averaged metadynamics. J. Chem. Theory Comput. *9*, 5610–5617.

Campbell, I., and Humphries, M. (2011). Integrin structure, activation, and interactions. Cold Spring Harb. Perspect. Biol. *3*, http://dx.doi.org/10.1101/cshperspect.a004994.

Cavalli, A., Vendruscolo, M., and Paci, E. (2005). Comparison of sequence-based and structure-based energy functions for the reversible folding of a peptide. Biophys. J. *88*, 3158–3166.

Cavalli, A., Camilloni, C., and Vendruscolo, M. (2013). Molecular dynamics simulations with replica-averaged structural restraints generate structural ensembles according to the maximum entropy principle. J. Chem. Phys. *138*, 094112.

Dustin, M., and Springer, T. (1989). T-cell receptor cross-linking transiently stimulates adhesiveness through LFA-1. Nature *341*, 619–624.

Dustin, M., and Springer, T. (1999). Intercellular adhesion molecules (ICAMs). In Guidebook to the Extracellular Matrix and Adhesion Proteins, T. Kreis and R. Vale, eds. (Sambrook Tooze).

Essmann, U., Perera, L., Berkowitz, M., Darden, T., Lee, H., and Pedersen, L. (1995). A smooth particle mesh Ewald method. J. Chem. Phys. *103*, 8577–8593.

Fu, B., Sahakyan, A.B., Camilloni, C., Tartaglia, G.G., Paci, E., Caflisch, A., Vendruscolo, M., and Cavalli, A. (2014). ALMOST: an all atom molecular simulation toolkit for protein structure determination. J. Comput. Chem. *35*, 1101–1105.

Gaillard, T., Martin, E., San Sebastian, E., Cossío, F.P., Lopez, X., Dejaegere, A., and Stote, R.H. (2007). Comparative normal mode analysis of LFA-1 integrin I-domains. J. Mol. Biol. *374*, 231–249.

Hess, B., Kutzner, C., van der Spoel, D., and Lindahl, E. (2008). GROMACS 4: algorithms for highly efficient, load-balanced, and scalable molecular simulation. J. Chem. Theory Comput. *4*, 435–447.

Humphrey, W., Dalke, A., and Schulten, K. (1996). VMD: visual molecular dynamics. J. Mol. Graph. *14*, 33–38.

Humphries, J.D., Byron, A., and Humphries, M.J. (2006). Integrin ligands at a glance. J. Cell Sci. *119*, 3901–3903.

Hynes, R.O. (2002). Integrins: bidirectional, allosteric signaling machines. Cell *110*, 673–687.

Hynes, R.O. (2009). The extracellular matrix: not just pretty fibrils. Science *326*, 1216–1219.

Jin, M., Song, G., Carman, C.V., Kim, Y.-S., Astrof, N.S., Shimaoka, M., Wittrup, D.K., and Springer, T.A. (2006). Directed evolution to probe protein allostery and integrin I domains of 200,000-fold higher affinity. Proc. Natl. Acad. Sci. USA *103*, 5758–5763.

Jorgensen, W., Chandrasekhar, J., Madura, J., Impey, R., and Klein, M. (1983). Comparison of simple potential functions for simulating liquid water. J. Chem. Phys. *79*, 926–935.

Kabsch, W., and Sander, C. (2004). Dictionary of protein secondary structure: pattern recognition of hydrogen-bonded and geometrical features. Biopolymers *22*, 2577–2637.

Kishimoto, T., Larson, R., Corbi, A., Dustin, M., Staunton, D., and Springer, T. (1988). Leukocyte Integrins, Paper presented at: Structure, Function and Regulation of Molecules Involved in Leukocyte Adhesion (Titisee, Germany: Springer).

Kohlhoff, K.J., Robustelli, P., Cavalli, A., Salvatella, X., and Vendruscolo, M. (2009). Fast and accurate predictions of protein NMR chemical shifts from interatomic distances. J. Am. Chem. Soc. *131*, 13894–13895.

Kriwacki, R., Legge, G., Hommel, U., Ramage, P., Chung, J., Tennant, L., Wright, P., and Dyson, J. (2000). Assignment of 1H, 13C and 15N resonances of the I-domain of human leukocyte function associated antigen-1. J. Biomol. NMR *16*, 271–272.

Kukic, P., Camilloni, C., Cavalli, A., and Vendruscolo, M. (2014). Determination of the individual roles of the linker residues in the interdomain motions of calmodulin using NMR chemical shifts. J. Mol. Biol. *426*, 1826–1838.

Laio, A., and Parrinello, M. (2002). Escaping free-energy minima. Proc. Natl. Acad. Sci. USA *99*, 12562–12566.

Larson, R., and Springer, T. (1990). Structure and function of leukocyte integrins. Immunol. Rev. *114*, 181–217.

Last-Barney, K., Davidson, W., Cardozo, M., Frye, L., Grygon, C., Hopkins, J., Jeanfavre, D., Pav, S., Qian, C., Stevenson, J., et al. (2001). Binding site elucidation of hydantoin-based antagonists of LFA-1 using multidisciplinary technologies: evidence for the allosteric inhibition of a protein-protein interaction. J. Am. Chem. Soc. *123*, 5643–5650.

Legge, G., Kriwacki, R., Chung, J., Hommel, U., Ramage, P., Case, D., Dyson, J., and Wright, P. (2000). NMR solution structure of the inserted domain of human leukocyte function associated antigen-1. J. Mol. Biol. *295*, 1251–1264.

Leung, H.T.A., Kukic, P., Camilloni, C., Bemporad, F., Simone, A., Aprile, F.A., Kumita, J.R., and Vendruscolo, M. (2014). NMR characterization of the conformational fluctuations of the human lymphocyte function-associated antigen-1 I-domain. Protein Sci. *23*, 1596–1606.

Ley, K., Laudanna, C., Cybulsky, M.I., and Nourshargh, S. (2007). Getting to the site of inflammation: the leukocyte adhesion cascade updated. Nat. Rev. Immunol. *7*, 678–689.

Losonczi, J., Andrec, M., Fischer, M., and Prestegard, J. (1999). Order matrix analysis of residual dipolar couplings using singular value decomposition. J. Magn. Reson. *138*, 334–342.

Lu, C., Shimaoka, M., Ferzly, M., Oxvig, C., Takagi, J., and Springer, T. (2001a). An isolated, surface-expressed I domain of the integrin αLβ2 is sufficient for strong adhesive function when locked in the open conformation with a disulfide bond. Proc. Natl. Acad. Sci. USA *98*, 2387–2392.

Lu, C., Shimaoka, M., Zang, Q., Takagi, J., and Springer, T. (2001b). Locking in alternate conformations of the integrin αLβ2 I domain with disulfide bonds reveals functional relationships among integrin domains. Proc. Natl. Acad. Sci. USA *98*, 2393–2398.

Luo, B.-H., Carman, C., and Springer, T. (2007). Structural basis of integrin regulation and signaling. Ann. Rev. Immunol. *25*, 619–647.

Marinelli, F., Pietrucci, F., Laio, A., and Piana, S. (2009). A kinetic model of trp-cage folding from multiple biased molecular dynamics simulations. PLoS Comput. Biol. *5*, e1000452.

Piana, S., and Laio, A. (2007). A bias-exchange approach to protein folding. J. Phys. Chem. B *111*, 4553–4559.

Pitera, J.W., and Chodera, J.D. (2012). On the use of experimental observations to bias simulated ensembles. J. Chem. Theory Comput. *8*, 3445–3451.

Robustelli, P., Kohlhoff, K., Cavalli, A., and Vendruscolo, M. (2010). Using NMR chemical shifts as structural restraints in molecular dynamics simulations of proteins. Structure *18*, 923–933.

Roux, B., and Weare, J. (2013). On the statistical equivalence of restrained-ensemble simulations with the maximum entropy method. J. Chem. Phys. *138*, 084107.

Shattil, S.J., Kim, C., and Ginsberg, M.H. (2010). The final steps of integrin activation: the end game. Nat. Rev. Mol. Cell Biol. *11*, 288–300.

Shen, Y., and Bax, A. (2010). SPARTA+: a modest improvement in empirical NMR chemical shift prediction by means of an artificial neural network. J. Biomol. NMR *48*, 13–22.

Shimaoka, M., Lu, C., Palframan, R., von Andrian, U., McCormack, A., Takagi, J., and Springer, T. (2001). Reversibly locking a protein fold in an active conformation with a disulfide bond: integrin αL I domains with high affinity and antagonist activity. Proc. Natl. Acad. Sci. USA *98*, 6009–6014.

Shimaoka, M., Takagi, J., and Springer, T. (2002). Conformational regulation of integrin structure and function. Annu. Rev. Biophys. Biomol. Struct. *31*, 485–516.

Shimaoka, M., Xiao, T., Liu, J.-H., Yang, Y., Dong, Y., Jun, C.-D., McCormack, A., Zhang, R., Joachimiak, A., Takagi, J., et al. (2003). Structures of the alpha L I domain and its complex with ICAM-1 reveal a shape-shifting pathway for integrin regulation. Cell *112*, 99–111.

Sugita, Y., and Okamoto, Y. (1999). Replica-exchange molecular dynamics method for protein folding. Chem. Phys. Lett. *314*, 141–151.

Takada, Y., Ye, X.J., and Simon, S. (2007). The integrins. Genome Biol. *8*, 215.

Tribello, G.A., Bonomi, M., Branduardi, D., Camilloni, C., and Bussi, G. (2014). PLUMED 2: new feathers for an old bird. Comput. Phys. Commun. *185*, 604–613.

Weitz-Schmidt, G., Welzenbach, K., Brinkmann, V., Kamata, T., Kallen, J., Bruns, C., Cottens, S., Takada, Y., and Hommel, U. (2001). Statins selectively inhibit leukocyte function antigen-1 by binding to a novel regulatory integrin site. Nat. Med. *7*, 687–692.

Xie, C., Zhu, J., Chen, X., Mi, L., Nishida, N., and Springer, T. (2010). Structure of an integrin with an alphaI domain, complement receptor type 4. EMBO J. *29*, 669–679.

Zhang, H., Liu, J.H., Yang, W., Springer, T., Shimaoka, M., and Wang, J.H. (2009). Structural basis of activation-dependent binding of ligand-mimetic antibody AL-57 to integrin LFA-1. Proc. Natl. Acad. Sci. USA *106*, 18345–18350.

Structure, Volume *23*

# Supplemental Information

# Structure and Dynamics of the Integrin LFA-1 I-Domain in the Inactive State Underlie its Inside-Out/ Outside-In Signaling and Allosteric Mechanisms

**Predrag Kukic, Hoi Tik Alvin Leung, Francesco Bemporad, Francesco A. Aprile, Janet R. Kumita, Alfonso De Simone, Carlo Camilloni, and Michele Vendruscolo**

## Supplemental Figures

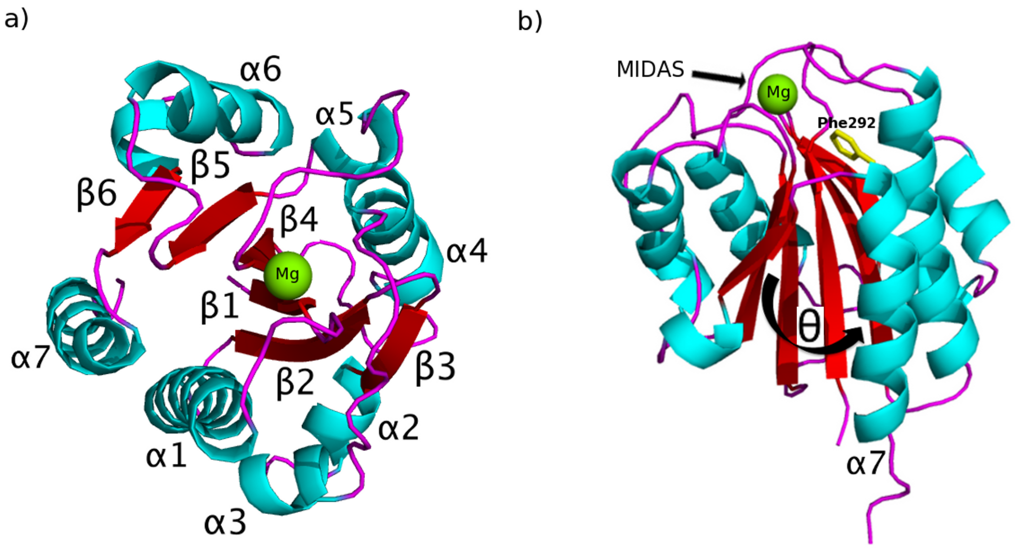


**Figure S1**, related to Figure 1. Secondary structure of the LFA-I domain. a) Cartoon view of the I-domain secondary structure elements taken from the low affinity (LA) state structure PBD ID: 1LFA. b) Definition of the angle θ between α-helix 7 and the hydrophobic core of the I-domain. The side chain of residue Phe292 in the LA state is locked inside the hydrophobic pocket that connects α-helix 7 and MIDAS, the so-called ‘ratchet’ pocket. In the intermediate affinity (IA) and high affinity (HA) states, the side chain of Phe292 is displaced from the ratchet pocket, which unlocks α-helix 7 and enables its movement along the side of the I-domain.

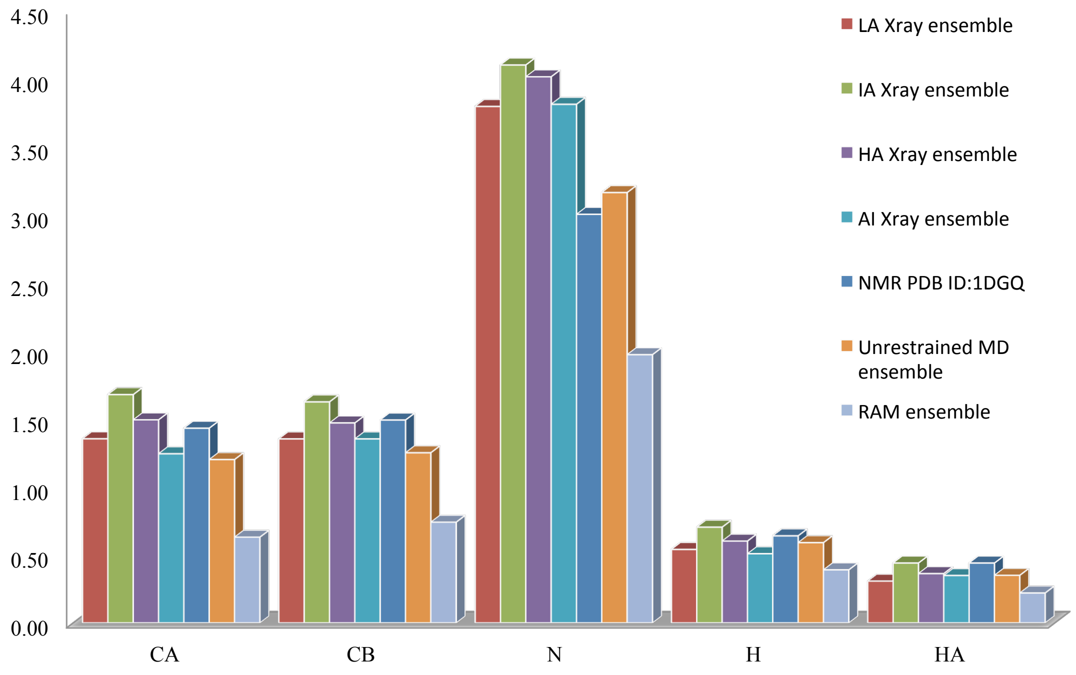


**Figure S2**, related to Figure 1. Bar chart representation related to Table S1.

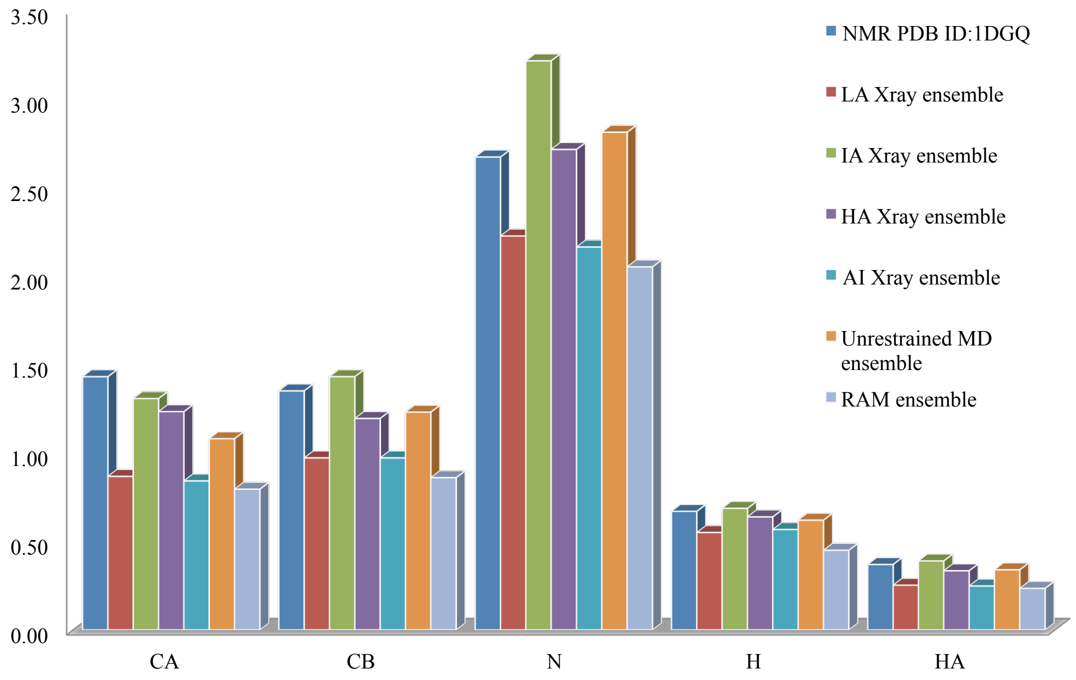


**Figure S3**, related to Figure 1. Bar chart representation related to Table S2.

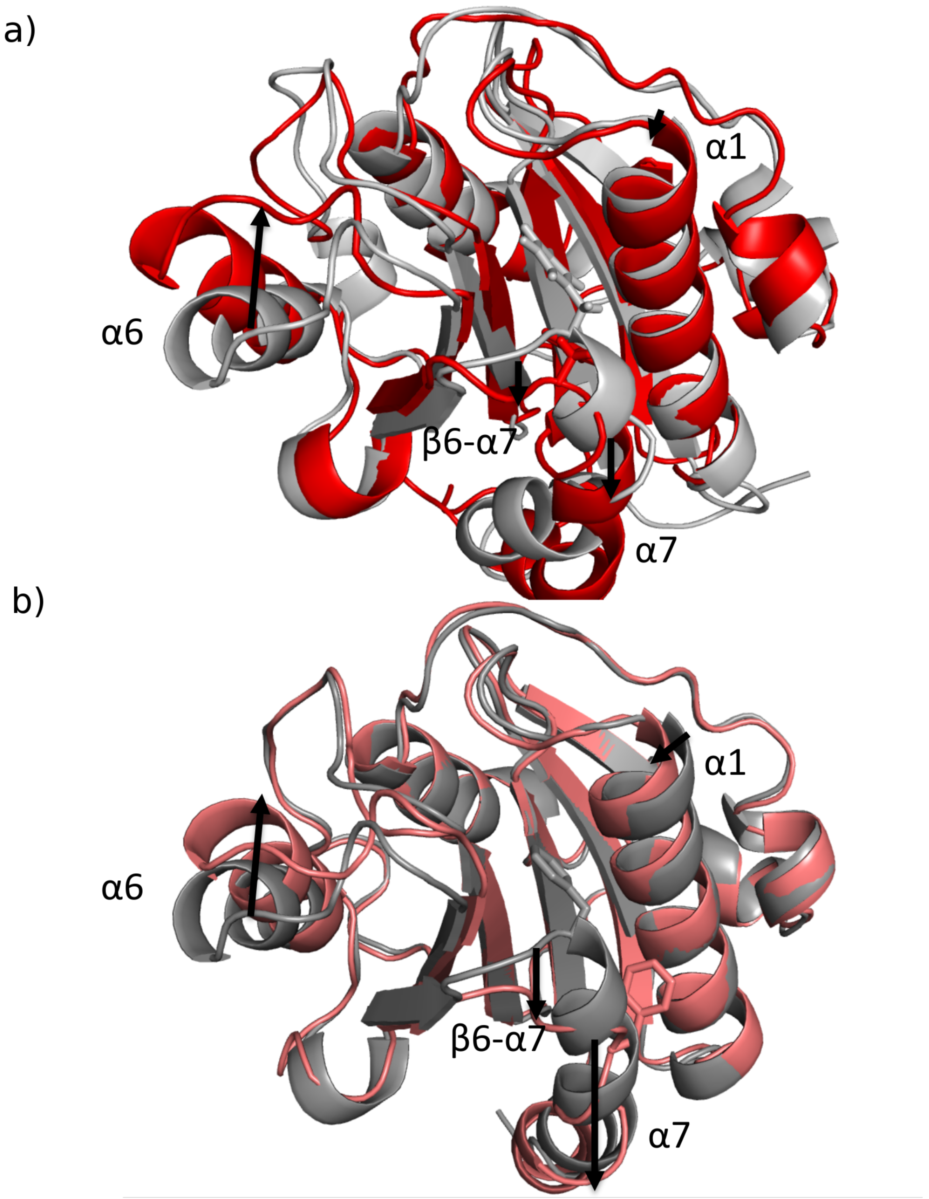


**Figure S4**, related to Figure 3. a) Superposition of the LA-like (gray) and IA-like states (red) from the RAM ensemble. Two representative structures with the lowest free energies in the RAM ensemble are superimposed using the hydrophobic core of the I-domain (residues 143-266). The arrows denote areas with the larges conformational differences: α-helix 7, loop between α-helix 7 and β-sheet 6, α-helices 1 and 6. b) Superposition of the representative crystallographic structures of the WT LFA in the LA (PDB ID: 3F74 depicted in gray) and HA states (PDB ID: 3TCX, depicted in light red). The areas of conformational differences are equivalent to those from the LA-like and IA-like structures.

## Supplemental Tables

**Table S1**, related to Figure 1**.** Chemical shift-based comparison between the ensembles of crystallographic structures of LFA in the LA, IA, HA and AI states, the NOESY-derived structure PDB ID: 1DGQ, the unrestrained MD ensemble and the RAM ensemble. The comparison is made in terms of RMSD (ppm) between the experimentally measured chemical shifts and the values back-calculated from the ensembles using the CamShift method (Kohlhoff et al., 2009).

| ensemble/chemical shifts | Cα | Cβ | N | $H_N$ | Hα |
|---|---|---|---|---|---|
| NMR PDB ID:1DGQ | 1.4 | 1.5 | 3.0 | 0.6 | 0.4 |
| LA Xray ensemble | 1.3 | 1.3 | 3.8 | 0.5 | 0.3 |
| IA Xray ensemble | 1.7 | 1.6 | 4.1 | 0.7 | 0.4 |
| HA Xray ensemble | 1.5 | 1.5 | 4.0 | 0.6 | 0.4 |
| AI Xray ensemble | 1.2 | 1.4 | 3.8 | 0.5 | 0.3 |
| Unrestrained MD ensemble | 1.2 | 1.3 | 3.2 | 0.6 | 0.4 |
| RAM ensemble | 0.6 | 0.7 | 2.0 | 0.4 | 0.2 |

**Table S2**, related to Figure 1**.** Chemical shift-based comparison between the ensembles of crystallographic structures of LFA in the LA, IA, HA and AI states, the NOESY-derived structure PDB ID: 1DGQ, the unrestrained MD ensemble and the RAM ensemble. The comparison is made in terms of RMSD (ppm) between the experimentally measured chemical shifts and the values back-calculated from the ensembles using SPARTA+ (Shen and Bax, 2010).

| ensemble/chemical shifts | Cα | Cβ | N | $H_N$ | Hα |
|---|---|---|---|---|---|
| NMR PDB ID:1DGQ | 1.4 | 1.4 | 2.7 | 0.7 | 0.4 |
| LA Xray ensemble | 0.9 | 1.0 | 2.2 | 0.6 | 0.3 |
| IA Xray ensemble | 1.3 | 1.4 | 3.2 | 0.7 | 0.4 |
| HA Xray ensemble | 1.2 | 1.2 | 2.7 | 0.6 | 0.3 |
| AI Xray ensemble | 0.9 | 1.0 | 2.2 | 0.6 | 0.3 |
| Unrestrained MD ensemble | 1.1 | 1.2 | 2.8 | 0.6 | 0.3 |
| RAM ensemble | 0.8 | 0.9 | 2.0 | 0.5 | 0.2 |

## Supplemental References

Kohlhoff, K.J., Robustelli, P., Cavalli, A., Salvatella, X., and Vendruscolo, M. (2009). Fast and accurate predictions of protein NMR chemical shifts from interatomic distances. J Am Chem Soc *131*, 13894-13895.

Shen, Y., and Bax, A. (2010). Sparta+: A modest improvement in empirical NMR chemical shift prediction by means of an artificial neural network. J Biomol NMR *48*, 13-22.
